# Risk factors for multimorbidity of cardiovascular diseases in the prospective Million Women Study

**DOI:** 10.1136/openhrt-2025-003950

**Published:** 2026-05-21

**Authors:** Jae Won Suh, Sarah Floud, Gillian K Reeves, Benjamin J Cairns, F Lucy Wright

**Affiliations:** 1 Research Department of Clinical, Educational, and Health Psychology, University College London, London, UK; 2 Cancer Epidemiology Unit, Nuffield Department of Population Health, University of Oxford, Oxford, UK; 3 Our Future Health, London, UK; 4 Applied Health Research Unit, Nuffield Department of Population Health, Oxford University, Oxford, UK

**Keywords:** CORONARY ARTERY DISEASE, EPIDEMIOLOGY, Electronic Health Records

## Abstract

**Objective:**

Cardiovascular diseases often occur together, but little is known about what increases the risk of developing cardiovascular multimorbidity (CVM), particularly in women. This study investigated the associations of cardiovascular risk factors with CVM incidence in UK women.

**Methods:**

1.3 million women aged 50–64 years were recruited into the Million Women Study in 1996–2001. Women reported information on demographic and cardiovascular risk factors (weight, height, smoking, alcohol consumption, physical activity and treatment for high blood pressure, diabetes and high blood cholesterol) at baseline. Using linked hospital admission and death records, each participant was followed for 19 subtypes of incident cardiovascular disease. Outcomes were CVM (having ≥2 of 19 selected cardiovascular disease diagnoses), complex CVM (having ≥4 diagnoses), and pairs of the four most common individual cardiovascular diseases.

**Results:**

In multivariable adjusted models, obesity, current smoking and treatment for diabetes or hypertension were each independently associated with a 2–3 times higher risk of incident CVM. Severe obesity, heavy smoking and diabetes were associated with 3–4 times higher risks of complex CVM and most CVM pairs. The strongest relationships were for severe obesity, which was associated with a fivefold higher risk of developing both atrial fibrillation and heart failure together (compared with healthy body mass index), and for diabetes, which was associated with a fivefold higher risk of developing both ischaemic heart disease and heart failure together. There was little evidence of strong associations of alcohol consumption or physical activity with most CVM outcomes.

**Conclusions:**

In middle-aged women, known cardiovascular risk factors including smoking and obesity were associated with substantially higher risks of CVM, with stronger associations for severe obesity, heavy smoking and diabetes, and certain combinations of cardiovascular disease subtypes. Targeted management of these risk factors for secondary prevention may reduce progression to CVM, potentially with greater benefits in those at risk for complex CVM.

**WHAT IS ALREADY KNOWN ON THIS TOPIC**

- Patients can often have more than one cardiovascular disease (CVD) diagnosis, yet few studies have investigated how established CVD risk factors relate to the risk of incident cardiovascular multimorbidity (CVM), defined here as co-occurring cardiovascular diagnoses.

**WHAT THIS STUDY ADDS**

- This study provides estimates of risk factor associations with incident CVM in over a million UK women. Obesity, current smoking and treatment for hypertension or diabetes were associated with a 2–3 times higher risk of CVM, and risks for complex CVM were somewhat larger in magnitude. Some associations for pairs of CVDs were especially strong; for example, severe obesity was associated with over 5 times the risk of developing both atrial fibrillation and heart failure together.

**HOW THIS STUDY MIGHT AFFECT RESEARCH, PRACTICE OR POLICY**

- Effective management of known risk factors in patients with either a single CVD diagnosis or existing CVM may reduce the occurrence of subsequent CVDs and therefore reduce the burden of CVM. Strong associations observed for specific risk factors and combinations of CVDs suggest potential for more personalised strategies for prevention of CVM.

## Introduction

 The co-occurrence of multiple long-term conditions in one individual (ie, multimorbidity) is becoming more common due to population ageing and better treatment of, and improved survival from, chronic diseases. One of the most common types of multimorbidity is the co-occurrence of ≥2 cardiovascular diseases (CVDs)^1^; we define this as cardiovascular multimorbidity (CVM). CVM is associated with higher mortality, lower quality of life, higher healthcare costs and complications in healthcare management.^2^,^4^ In an earlier study, we estimated that over 60% of middle-aged UK women with a CVD will develop at least one other CVD subtype by approximately 80 years of age.^5^ However, reliable evidence on risk factors for the development of CVM is scarce, and previous studies have focused on a limited number of individual CVDs.^6 7^ In this study, we used data from a cohort of 1.3 million UK women to investigate the prospective associations of cardiovascular risk factors with the development of CVM.

## Methods

### Study design and participants

The Million Women Study is a population-based cohort study of 1.3 million UK women. Details of the study have been reported elsewhere.^8 9^ Briefly, women aged 50–64 years were recruited between 1996 and 2001 through the National Breast Screening Programme. Each participant was linked by their unique identification number to National Health Service Central Registers, providing data on cancer registrations, hospitalisations and deaths. Disease diagnoses and causes of death were coded using the International Classification of Diseases 10th edition (ICD-10).

### Outcomes

19 CVD subtypes were selected by clinical importance and number of cases, predominantly from chapter IX (Diseases of the circulatory system) of the ICD-10 (online supplemental appendix A). The main outcome was CVM, defined as having ≥2 diagnoses from these CVD subtypes. Secondary outcomes were six pairs of CVDs comprised of the four most common CVD subtypes found in CVM (ischaemic heart disease, atrial fibrillation, heart failure and stroke). Complex CVM (≥4 CVDs) was studied as a more serious form of CVM.

An incident CVD was defined as the first recorded event since study recruitment. An event was identified by a record with a relevant ICD-10 code in any diagnosis field in hospital admission records, or as the underlying cause of death. The date of CVM was defined as the date of the second diagnosis. This could have been the same as the date of the first diagnosis if ≥2 CVDs were first recorded on the same day. The date of complex CVM was the date of the fourth diagnosis, which similarly could be the same as the date of any or all of the previous diagnoses. The date of a CVM pair was the later of the dates of record of the two diseases in a pair (which could be the same date).

### Risk factors

Risk factors investigated in these analyses were: (1) anthropometric factors, namely body mass index (BMI; <20, 20–24, 25–29, 30–34, ≥35 kg/m^2^) and height (<155, 155–159, 160–164, 165–169, ≥170 cm); (2) lifestyle and behavioural factors, namely cigarette smoking (never, past, <15 per day, ≥15 per day), alcohol consumption (1–2, 3–6, 7–14, ≥15 units per week) and strenuous physical activity (none, up to once per week, 2–3 times per week, ≥4 times per week); and (3) reported treatment for hypertension (yes, no), diabetes (yes, no) or high blood cholesterol (yes, no) at study recruitment. All risk factor data were collected through the self-reported questionnaire completed at recruitment (https://www.ceu.ox.ac.uk/research/million-women-study-1/questionnaires).

### Statistical analyses

After standard Million Women Study exclusions (online supplemental appendix B) and exclusion of women with any CVD or cancer (except non-melanoma skin cancer) at baseline, a total of 1 244 294 women were included in analyses.

Cox proportional hazards regression with attained age as the underlying time variable was used to calculate HRs and 95% CIs for the CVM outcomes (CVM and complex CVM) in relation to the selected risk factors. Floated CIs (FCIs) were presented in the forest plots, while conventional CIs were given in the text for explicit comparisons between two groups; FCIs permit comparisons across all categories without specifying a reference.^10^ Person-years were calculated from the date of recruitment until the date of the earliest of: the first hospital admission for CVM; the first recorded cancer diagnosis in hospital admission or cancer registration; death; emigration or other loss to follow-up; or the end of follow-up. Follow-up was censored at the first recorded cancer diagnosis to avoid possible iatrogenic and indirect associations, since cancer and CVDs share risk factors, and cancer treatment can affect CVD risk factors and increase CVD risk.^11 12^ Follow-up ended on 31 December 2019. Fewer than 1.5% of women were lost to follow-up. For approximately 5% of women recruited in England before 1 April 1997, follow-up began on that date due to lack of hospital admission data.

All regression models were stratified by age at recruitment (<53, 53–55, 56–58, 59–61, 62–64, ≥65 years), to account for time in study and cohort effects, and by recruitment region (Oxford, East Anglia, South West, Thames, West Midlands, North York, Trent, North West (Mersey), North West (Manchester/Lancaster), Scotland). Multivariable adjusted models were adjusted for BMI, height, smoking and strenuous physical activity (as above) and for alcohol consumption (rarely/never, 1–2, 3–6, 7–14, ≥15 units per week), socioeconomic deprivation (fifths of Townsend index^13^), educational qualifications (none, technical qualifications, secondary school, tertiary (college or university)), parity (nulliparous, 1–2, ≥3 full-term pregnancies) and ever use of menopausal hormone therapy (yes or no). Multivariable adjusted HRs for treatment for hypertension, high cholesterol and diabetes were not adjusted for BMI, since BMI may modify the relationships between these factors and some CVDs.^14^ Estimates for other risk factors were likewise not adjusted for treatment for hypertension, high cholesterol and diabetes, as they were likely mediators in the relationships between the examined risk factors and CVDs. For each adjustment variable, missing values (<6% for each variable) were assigned to a separate category (online supplemental appendix C).

When alcohol consumption was the risk factor of interest, women who drank less than one alcoholic drink per week (women who do not drink alcohol) were excluded. Over 85% of women who do not drink alcohol at recruitment were women who quit drinking alcohol,^15^ and stopped for unknown reasons which may have included poor cardiovascular health, potentially biasing associations between alcohol consumption and CVM.

Heterogeneity in HRs across the categories of each risk factor were tested using the likelihood ratio test (α=0.05). The false discovery rate was controlled using the Benjamini-Hochberg procedure.^16^ Log-log plots were assessed to check the proportional hazards assumption, which was found to be satisfied in all models.

We used the relative change in the likelihood ratio (LR) statistic from the fully adjusted model, compared with the minimally adjusted model, as an indication of the extent to which the relationships between risk factors and CVM outcomes were accounted for by multivariable adjustment.^17 18^ Relative changes in LR statistics were calculated as $\left(1-\frac{LR\chi_{a\mathrm{d}j}^{2}}{LR\chi_{min}^{2}}\right)\cdot 100$, where $LR\chi_{min}^{2}$ is the LR statistic of the minimally adjusted model and $LR\chi_{adj}^{2}$ is the LR statistic of the additionally adjusted model (‘attenuation in LR statistics’ or ‘$\chi_{attenuation}^{2}$’ in the text and figures). For the purposes of this analysis, we deemed associations for which this attenuation was large (eg, >80%) after multivariable adjustment to be more likely to be due to residual confounding (since the covariates were measured with error), than those for which the corresponding attenuation was small (eg, <20%).

Possible reverse causation bias was assessed in sensitivity analyses: (1) excluding the first 5 years of follow-up, (2) excluding both the first 3 years of follow-up and women who did not report good or excellent self-rated health (which may indicate subclinical disease or other unrecorded illness that influences behaviour) and (3) including women who do not drink alcohol in the analyses of alcohol consumption.

All analyses were conducted in Stata V.17 (StataCorp, College Station, Texas, USA) and R Statistical Software (V.4.2.1; R Core Team 2021).

## Results

### Baseline characteristics

The study included 1 244 294 women with an average age at recruitment of 56.5 (SD 4.8) years; 20.4% were women who smoke currently, mean BMI was 26.1 (SD 4.6) kg/m^2^ and 19.2% lived in the most deprived areas (table 1). Women diagnosed with a higher number of CVD subtypes were, on average, older at recruitment, more likely to smoke, have a higher BMI and live in the most deprived areas than those with fewer CVD subtypes. Those who developed incident CVM were much more likely to report being treated for hypertension (13.1% vs 25.1%), diabetes (1.6% vs 5.5%) and high blood cholesterol (2.3% vs 4.7%) than those who did not develop CVM.

**Table 1:** Baseline characteristics in all women and by their incident CVM status at the end of follow-up

CVM status	No CVM		CVM			All women
Number of incident CVDs	0 disease	1 disease	2 diseases	3 diseases	4+ diseases	
Women, n	922 584	174 063	72 777	36 313	38 557	1 244 294
Age at recruitment, mean (SD), years	55.9 (4.6)	57.5 (4.9)	58.5 (5.1)	59.1 (5.1)	59.8 (5.1)	56.5 (4.8)
Lifestyle and anthropometric factors						
Women who smoke currently, % (n)	18.6 (161 594)	24.5 (40 127)	26.3 (17 950)	26.9 (9146)	28.9 (10 439)	20.4 (239 256)
Women who drink alcoholic beverages, % (n)	67.1 (614 793)	60.7 (104 630)	58.5 (42 141)	56.9 (20 446)	55.1 (20 988)	65.0 (802 998)
No strenuous physical activity, % (n)	58.8 (524 211)	64.1 (106 821)	65.9 (45 787)	66.9 (23 102)	68.2 (24 878)	60.4 (724 799)
Body mass index, mean (SD), kg/m^2^	25.9 (4.4)	26.7 (4.9)	26.9 (5.2)	27.3 (5.3)	27.6 (5.5)	26.1 (4.6)
Height, mean (SD), cm	162.1 (6.5)	162.0 (6.7)	162.1 (6.8)	162.1 (6.8)	162.1 (6.9)	162.1 (6.6)
Socioeconomic factors						
Living in the most deprived areas, % (n)	17.7 (161 716)	22.4 (38 732)	23.8 (17 185)	25.4 (9164)	26.4 (10 113)	19.2 (236 910)
No educational qualifications, % (n)	40.5 (364 324)	48.6 (82 076)	51.0 (35 961)	53.6 (18 794)	55.7 (20 702)	43.1 (521 857)
Reproductive factors						
Parity, mean (SD)	2.1 (1.2)	2.2 (1.3)	2.3 (1.3)	2.3 (1.4)	2.4 (1.4)	2.2 (1.2)
Ever use of HRT, % (n)	50.3 (459 235)	52.2 (89 561)	50.4 (36 140)	48.1 (17 170)	45.7 (17 283)	50.4 (619 389)
Healthcare factors						
Self-reported treatment for hypertension, % (n)	12.0 (111 023)	18.6 (32 443)	22.0 (15 975)	26.1 (9455)	30.1 (11 607)	14.5 (180 503)
Self-reported treatment for diabetes mellitus, % (n)	1.3 (12 416)	3.0 (5179)	4.2 (3078)	5.6 (2037)	7.9 (3035)	2.1 (25 745)
Self-reported treatment for high cholesterol, % (n)	2.1 (19 455)	3.5 (6082)	4.2 (3048)	4.7 (1688)	5.6 (2174)	2.6 (32 447)

CVM, cardiovascular multimorbidity; HRT, hormone replacement therapy.

### Risk factors for CVM and complex CVM

**Figure 1:**
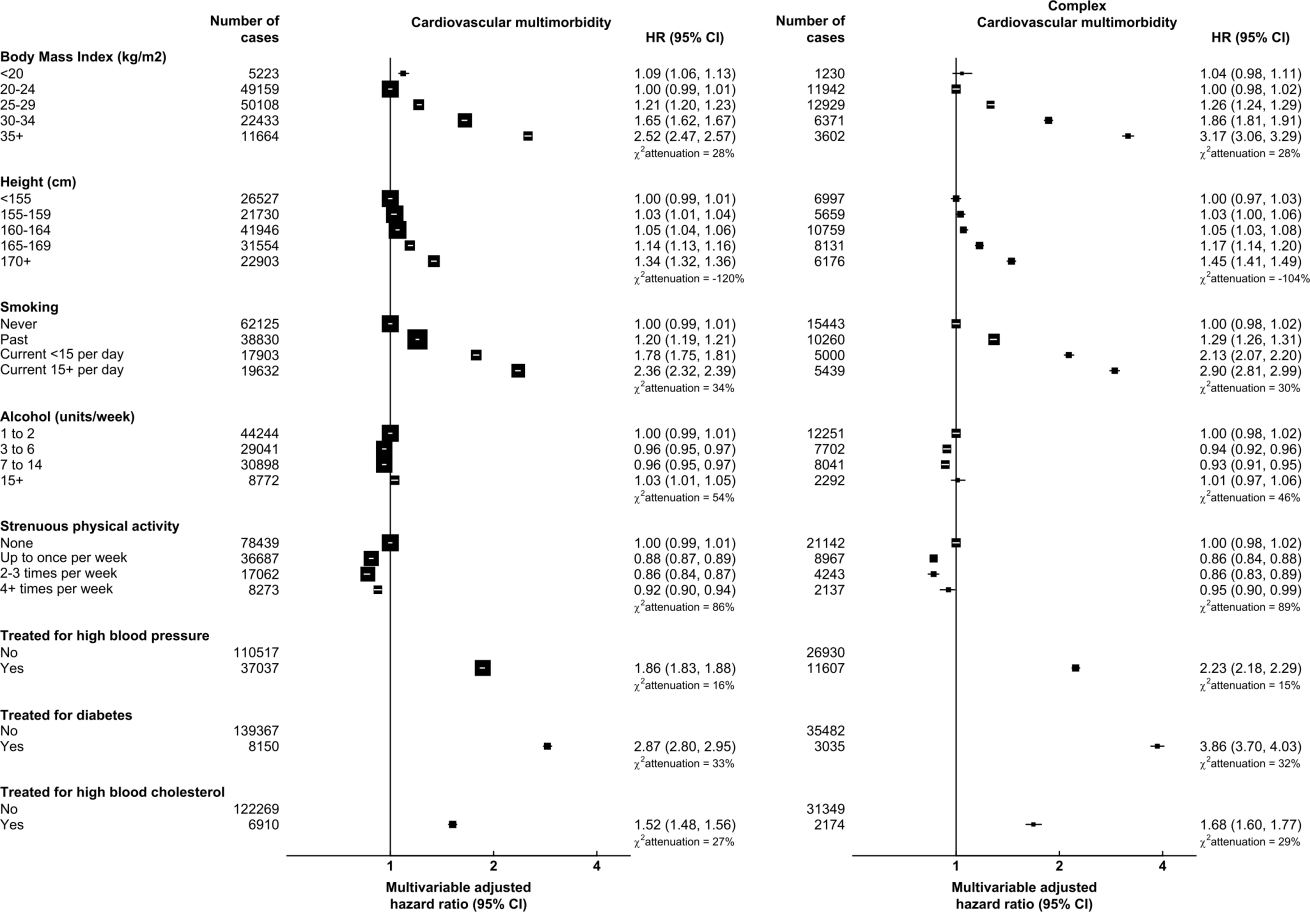
Relative risks of incident cardiovascular multimorbidity in relation to cardiovascular risk factors. Minimally adjusted models were stratified by age at recruitment and area of residence only. Multivariable adjusted models were additionally adjusted for area deprivation, educational attainment, physical activity, smoking, alcohol consumption, height, body mass index, parity and ever use of menopausal hormone therapy. Models for treatment for high blood pressure, diabetes and high blood cholesterol were not adjusted for body mass index. The size of the square boxes was inversely proportional to the SE of the effect estimate. $\chi_{attenuation}^{2}$ was calculated as the relative change in the likelihood-ratio $\chi^{2}$statistic between the minimally and multivariable adjusted models. Floated CIs were displayed where more than two groups were compared.

Figure 1 displays multivariable adjusted HRs and 95% FCIs for incident CVM and complex CVM for each risk factor. All associations were statistically significant after controlling for the false discovery rate (p for heterogeneity <0.001). Obesity was associated with a higher risk of CVM: the HRs for CVM were 1.65 (95% CI 1.62 to 1.67) in obese women (BMI 30–34 kg/m^2^) and 2.52 (2.47–2.57) in severely obese women (BMI ≥35 kg/m^2^), compared with those with a healthy BMI (20–24 kg/m^2^). Being taller was associated with a higher risk of CVM: the HR for CVM was 1.34 (1.32–1.36) in the tallest women (≥170 cm) compared with the shortest (<155 cm).

Current smoking was associated with twice the risk of CVM than never smoking. The HR of CVM for women who smoke currently and smoked <15 cigarettes per day compared with women who have never smoked was 1.78 (1.75–1.81), and for women who smoke heavily (≥15 cigarettes per day) was 2.36 (2.32–2.39). Other behavioural risk factors, alcohol consumption and physical activity, showed little or no association with CVM.

Treatment for high blood pressure, diabetes mellitus and high blood cholesterol were each associated with 1.86 (1.83–1.88), 2.87 (2.80–2.95) and 1.52 (1.48–1.56) times higher risk of CVM, respectively.

Risk factor associations for complex CVM (ie, developing ≥4 CVD subtypes) were similar in shape and somewhat larger in magnitude than those for CVM (≥2 CVD subtypes). For instance, being treated for diabetes was associated with a higher risk of complex CVM (3.86, 3.70–4.03) than CVM (2.87, 2.80–2.95).

### Risk factors for pairs of common CVD subtypes

**Figure 2:**
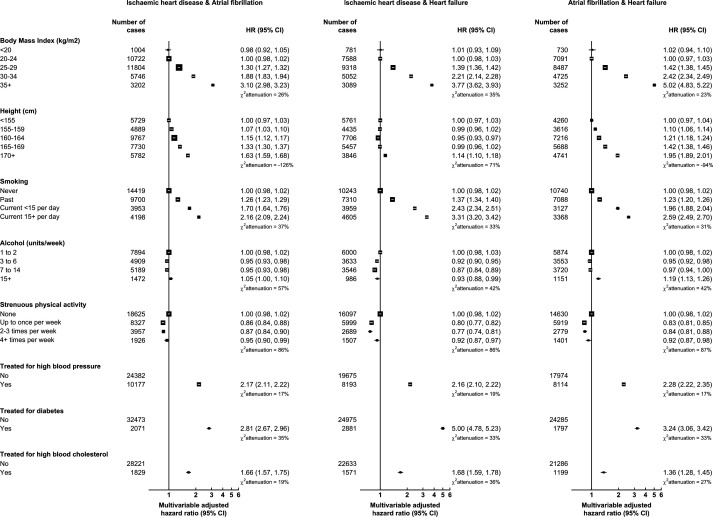
Relative risks for pairs of common heart diseases in relation to cardiovascular risk factors. Minimally adjusted models were stratified by age at recruitment and area of residence only. Multivariable adjusted models were additionally adjusted for area deprivation, educational attainment, physical activity, smoking, alcohol consumption, height, body mass index, parity and ever use of menopausal hormone therapy. Models for treatment for high blood pressure, diabetes and high blood cholesterol were not adjusted for body mass index. The size of the square boxes was inversely proportional to the SE of the effect estimate. $\chi_{attenuation}^{2}$ was calculated as the relative change in the likelihood-ratio $\chi^{2}$statistic between the minimally and multivariable adjusted models. Floated CIs were displayed where more than two groups were compared.

**Figure 3:**
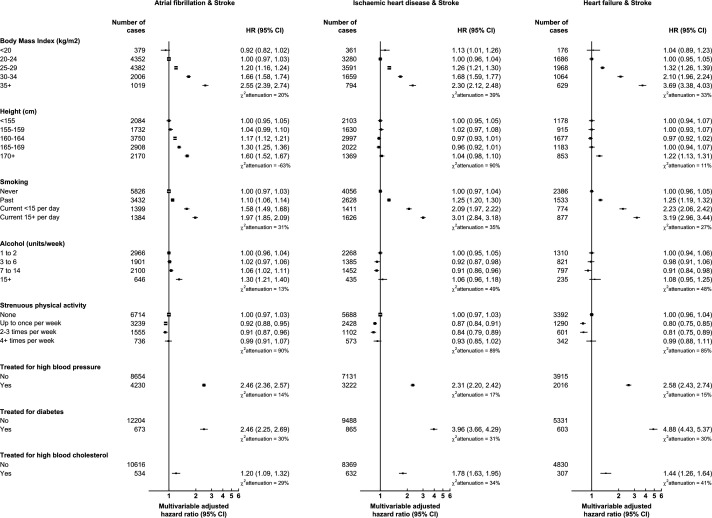
Relative risks for common cardiovascular multimorbidity pairs including stroke in relation to cardiovascular risk factors. Minimally adjusted models were stratified by age at recruitment and area of residence only. Multivariable adjusted models were additionally adjusted for area deprivation, educational attainment, physical activity, smoking, alcohol consumption, height, body mass index, parity and ever use of menopausal hormone therapy. Models for treatment for high blood pressure, diabetes and high blood cholesterol were not adjusted for body mass index. The size of the square boxes was inversely proportional to the SE of the effect estimate. $\chi_{attenuation}^{2}$ was calculated as the relative change in the likelihood-ratio $\chi^{2}$statistic between the minimally and multivariable adjusted models. Floated CIs were displayed where more than two groups were compared.

Risk factor associations for the selected CVM pairs (figures 2,3) were often similar in shape and magnitude to associations for complex CVM, and again larger in magnitude than those for CVM.

The CVM pairs involving heart failure (paired with ischaemic heart disease, atrial fibrillation or stroke) had larger relative risks associated with BMI than did other CVM outcomes. The largest relative risk associated with BMI was observed for the CVM pair of atrial fibrillation and heart failure (multivariable adjusted HR 5.02, 95% CI 4.83 to 5.22 in BMI ≥35 kg/m^2^ (severe obesity) vs 20–24 kg/m^2^ (healthy BMI), figure 2), followed by the pair of ischaemic heart disease and heart failure (3.77, 3.62–3.93, figure 2), and heart failure and stroke (3.69, 3.38–4.03, figure 3). Treatment for diabetes was also strongly associated with the CVM pair of ischaemic heart disease and heart failure (5.00, 4.78–5.23, figure 2) and the pair of heart failure and stroke (4.88, 4.43–5.37, figure 3).

Pairs involving atrial fibrillation also showed notable associations with height and (unlike CVM in general) alcohol consumption. The strongest association with height was for the CVM pair of atrial fibrillation and heart failure, for which women taller than 170 cm had an almost twice the risk compared with women shorter than 155 cm (HR 1.95, 1.89–2.01, figure 2). Though smaller in magnitude than associations with other risk factors, drinking ≥15 units of alcohol per week (vs 1–2 units) was also associated with the pairs of atrial fibrillation with both heart failure (1.19, 1.13–1.26, figure 2) and stroke (1.30, 1.21–1.40, figure 3).

### Attenuation in likelihood ratio test statistics

Attenuation in the LR statistics was small to moderate (up to ~50%) for most risk factors, suggesting that residual confounding is unlikely to explain all the multivariable adjusted associations (figures 1–3). The attenuation was larger, however, for physical activity and all CVM outcomes (85–90%), suggesting substantial potential for residual confounding after adjustment for factors measured with a degree of error.

In contrast, the relationships with height were often inflated after multivariable adjustment (eg, $\chi_{attenuation}^{2}$= −120% for CVM, figure 1), indicating that in minimally adjusted models, confounding masked stronger associations.

Estimates from the minimally adjusted models (stratified only by age and region at recruitment) are shown in online supplemental appendix D.

### Sensitivity analyses

Excluding the first 5 years of follow-up did not materially change the risk factor associations with CVM or complex CVM, while excluding both the first 3 years of follow-up and women who did not self-report good or excellent self-rated health slightly attenuated the associations with strenuous physical activity, suggesting there may be some bias attributable to reverse causation (table 2). As expected, the results of sensitivity analyses including women who do not drink alcohol, who may have stopped drinking alcohol due to poor cardiovascular health, showed potential reverse causation (online supplemental appendix E).

**Table 2:** Sensitivity analyses controlling for potential reverse causation bias in comparison to the main results

	Multivariable adjusted HR (95% CI)					
	Cardiovascular multimorbidity			Complex cardiovascular multimorbidity		
	Main analysis*	Sensitivity 1†	Sensitivity 2‡	Main analysis*	Sensitivity 1†	Sensitivity 2‡
BMI (kg/m^2^)						
<20	1.09 (1.06 to 1.13)	1.06 (1.02 to 1.10)	1.05 (1.00 to 1.11)	1.04 (0.98 to 1.11)	1.03 (0.94 to 1.12)	1.05 (0.94 to 1.17)
20–24	1.00 (referent)	1.00 (referent)	1.00 (referent)	1.00 (referent)	1.00 (referent)	1.00 (referent)
25–29	1.21 (1.20 to 1.23)	1.20 (1.18 to 1.22)	1.21 (1.18 to 1.24)	1.26 (1.23 to 1.30)	1.24 (1.20 to 1.29)	1.23 (1.18 to 1.29)
30–34	1.65 (1.62 to 1.68)	1.63 (1.59 to 1.67)	1.59 (1.54 to 1.64)	1.86 (1.80 to 1.92)	1.88 (1.79 to 1.97)	1.77 (1.66 to 1.88)
35+	2.52 (2.46 to 2.58)	2.48 (2.41 to 2.56)	2.36 (2.26 to 2.47)	3.17 (3.04 to 3.31)	3.18 (3.00 to 3.37)	2.98 (2.73 to 3.25)
Height (cm)						
<155	1.00 (referent)	1.00 (referent)	1.00 (referent)	1.00 (referent)	1.00 (referent)	1.00 (referent)
155–159	1.03 (1.01 to 1.05)	1.04 (1.01 to 1.06)	1.05 (1.01 to 1.09)	1.03 (0.99 to 1.07)	1.02 (0.96 to 1.07)	1.05 (0.97 to 1.13)
160–164	1.05 (1.03 to 1.07)	1.07 (1.04 to 1.09)	1.09 (1.05 to 1.12)	1.05 (1.02 to 1.09)	1.05 (1.00 to 1.10)	1.12 (1.05 to 1.19)
165–169	1.14 (1.12 to 1.16)	1.16 (1.13 to 1.19)	1.18 (1.15 to 1.22)	1.17 (1.13 to 1.21)	1.15 (1.09 to 1.21)	1.19 (1.12 to 1.28)
170+	1.34 (1.32 to 1.37)	1.37 (1.34 to 1.41)	1.41 (1.37 to 1.46)	1.45 (1.39 to 1.51)	1.45 (1.38 to 1.53)	1.56 (1.45 to 1.67)
Smoking						
Never	1.00 (referent)	1.00 (referent)	1.00 (referent)	1.00 (referent)	1.00 (referent)	1.00 (referent)
Past	1.20 (1.18 to 1.22)	1.16 (1.13 to 1.18)	1.16 (1.14 to 1.19)	1.29 (1.25 to 1.32)	1.22 (1.17 to 1.26)	1.25 (1.19 to 1.31)
Current <15 per day	1.78 (1.75 to 1.82)	1.71 (1.67 to 1.75)	1.70 (1.64 to 1.76)	2.13 (2.06 to 2.21)	2.04 (1.94 to 2.14)	2.08 (1.94 to 2.23)
Current 15+ per day	2.36 (2.31 to 2.40)	2.29 (2.23 to 2.34)	2.33 (2.25 to 2.42)	2.90 (2.79 to 3.00)	2.81 (2.67 to 2.96)	2.91 (2.71 to 3.13)
Alcohol (units per week)						
1–2	1.00 (referent)	1.00 (referent)	1.00 (referent)	1.00 (referent)	1.00 (referent)	1.00 (referent)
3–6	0.95 (0.93 to 0.97)	0.96 (0.93 to 0.98)	0.95 (0.92 to 0.98)	0.94 (0.90 to 0.97)	0.93 (0.89 to 0.98)	0.93 (0.87 to 0.99)
7–14	0.94 (0.92 to 0.96)	0.96 (0.94 to 0.98)	0.95 (0.92 to 0.98)	0.91 (0.88 to 0.95)	0.94 (0.89 to 0.99)	0.91 (0.86 to 0.97)
15+	1.01 (0.98 to 1.04)	1.05 (1.01 to 1.09)	1.02 (0.97 to 1.07)	0.99 (0.94 to 1.06)	1.05 (0.97 to 1.14)	0.97 (0.88 to 1.07)
Strenuous physical activity						
None	1.00 (referent)	1.00 (referent)	1.00 (referent)	1.00 (referent)	1.00 (referent)	1.00 (referent)
Up to once per week	0.88 (0.87 to 0.89)	0.91 (0.89 to 0.93)	0.95 (0.93 to 0.97)	0.86 (0.84 to 0.89)	0.91 (0.87 to 0.94)	0.92 (0.88 to 0.97)
2–3 times per week	0.86 (0.84 to 0.87)	0.90 (0.88 to 0.92)	0.95 (0.93 to 0.98)	0.86 (0.83 to 0.89)	0.93 (0.88 to 0.97)	0.99 (0.93 to 1.05)
4+ times per week	0.92 (0.89 to 0.94)	0.94 (0.91 to 0.97)	1.01 (0.97 to 1.05)	0.95 (0.90 to 0.99)	1.01 (0.95 to 1.08)	1.00 (0.92 to 1.08)
Being treated for high blood pressure						
No	1.00 (referent)	1.00 (referent)	1.00 (referent)	1.00 (referent)	1.00 (referent)	1.00 (referent)
Yes	1.86 (1.83 to 1.88)	1.75 (1.72 to 1.78)	1.79 (1.75 to 1.83)	2.23 (2.18 to 2.29)	2.07 (2.00 to 2.14)	2.17 (2.07 to 2.27)
Being treated for diabetes						
No	1.00 (referent)	1.00 (referent)	1.00 (referent)	1.00 (referent)	1.00 (referent)	1.00 (referent)
Yes	2.87 (2.80 to 2.95)	2.53 (2.44 to 2.62)	2.44 (2.30 to 2.59)	3.86 (3.70 to 4.03)	3.31 (3.10 to 3.54)	3.32 (3.00 to 3.67)
Being treated for high blood cholesterol						
No	1.00 (referent)	1.00 (referent)	1.00 (referent)	1.00 (referent)	1.00 (referent)	1.00 (referent)
Yes	1.52 (1.48 to 1.56)	1.38 (1.33 to 1.43)	1.42 (1.35 to 1.49)	1.68 (1.60 to 1.77)	1.44 (1.34 to 1.55)	1.65 (1.50 to 1.81)

Models were stratified by age at recruitment and area of residence and adjusted for area deprivation, educational attainment, physical activity, smoking, alcohol consumption, height, body mass index, parity and ever use of menopausal hormone therapy. Models for treatment for high blood pressure, diabetes and high blood cholesterol were not adjusted for body mass index.

* Main analysis=no exclusions or restrictions (N=1 244 294).

† Sensitivity 1=excluding the first 5 years of follow-up (N=1 016 030).

‡ Sensitivity 2=restricting to women who reported good or excellent health. Self-rated health was reported in the first resurvey questionnaire, on average 3 years after study recruitment. Therefore, Sensitivity 2 also excluded the first 3 years of follow-up in effect. (N=537 971).

BMI, body mass index.

## Discussion

### Key findings

In this study of 1.2 million UK women, several established CVD risk factors—obesity (vs healthy BMI), current smoking (vs never smoking) and treatment for hypertension or diabetes—were each associated with 2–3 times higher risks of CVM. Some risk factors also showed pronounced associations with specific pairs of co-occurring CVDs and with complex CVM (≥4 CVDs). For instance, severe obesity (BMI ≥35 kg/m^2^) was associated with over a fivefold risk of developing both atrial fibrillation and heart failure, while treatment for diabetes was also associated with an approximately fivefold risk of developing both stroke and heart failure. Severe obesity, heavy smoking and treatment for diabetes were associated with 3–4 times higher risks of complex CVM. Established cardiovascular risk factors are therefore important not only in individual CVDs but also in CVM.

### Findings in context

Previous prospective evidence on risk factors for CVM is limited. The most relevant are three studies on cardiometabolic multimorbidity, which they defined as developing at least two conditions from coronary heart disease, stroke and diabetes.^6 7 19^ We defined diabetes as a risk factor rather than an outcome, which limits the comparison with these studies, but all three found positive associations between BMI and cardiometabolic multimorbidity. These associations were slightly stronger than in our study, potentially due to the inclusion of diabetes, with its strong link to obesity.^20^ In one of the studies,^6^ relative risks for the association between BMI and vascular disease only (ie, coronary heart disease, stroke or both, but not diabetes) were of similar magnitudes to our estimates for CVM.

In our study, severe obesity was associated with a fivefold higher risk of developing both atrial fibrillation and heart failure together, representing the strongest association observed across all risk factor-outcome combinations. This finding is consistent with previous studies between obesity and both atrial fibrillation and heart failure individually, reporting approximately twofold higher risk of atrial fibrillation and twofold to threefold higher risk of heart failure among those with severe obesity compared with those with normal BMI.^21 22^ Severe obesity may disproportionately increase the risk of developing both conditions because obesity-related cardiac changes (such as epicardial fat deposition, left atrial enlargement, ventricular hypertrophy, and myocardial inflammation) predispose to both conditions simultaneously.^23 24^

To our knowledge, the relationship between height and CVM has not been examined previously. Nevertheless, prior studies have found associations between taller height and the development of several individual CVDs, including atrial fibrillation, venous thromboembolism and cardioembolic stroke.^25^,^27^ In particular, both standard observational and Mendelian randomisation studies have reported higher risks of atrial fibrillation in taller individuals, potentially reflecting differences in cardiac structure and electrophysiology, such as larger atrial size and differences in conduction pathways.^27^,^29^ Consistent with these findings, we observed stronger associations between height and CVM combinations that included atrial fibrillation.

Current smoking, physical inactivity and hypertension were also investigated in two of the three studies on cardiometabolic multimorbidity.^7 19^ Although each study comprised only a few hundred incident cases, associations with current smoking (around twice the risk) and with physical activity (no significant associations) were consistent with our findings. For hypertension, the Australian Longitudinal Study on Women’s Health found that self-reported diagnosis of hypertension was associated with approximately double the risk of cardiometabolic multimorbidity,^19^ while in the Whitehall II cohort, measured blood pressure ≥140/90 mm Hg or use of antihypertensive medication was associated with a 1.5 times higher risk.^7^

There were limited associations between physical activity and alcohol consumption with CVM outcomes in our study. This is broadly consistent with prior studies reporting heterogeneous relationships between these risk factors and individual CVDs, with generally modest effect sizes and mixed findings across some studies. For example, meta-analyses and Mendelian randomisation studies reported moderately protective associations of physical activity with ischaemic heart disease and stroke, but little or no association with atrial fibrillation.^30 31^ For alcohol consumption, Mendelian randomisation studies found little association with heart failure, atrial fibrillation and myocardial infarction but positive associations with stroke and peripheral artery disease.^32^,^35^ When CVDs with differing (and in some cases opposing) associations with a given risk factor are considered together, the overall association may be expected to appear weak or absent.

No other studies have reported on risk factors for complex multimorbidity exclusively comprised of CVDs, or on a broad range of pairs of CVDs. We found that women with less favourable cardiovascular risk factors are more likely to develop not only individual CVDs, as previously reported,^36^,^38^ but also CVM, CVM pairs specific to their risk factor profile and complex CVM. Moreover, associations with complex CVM and specific CVD pairs were stronger than with CVM. Complex CVM and some CVM pairs likely represent more severe phenotypes of CVD,^2^ with a greater burden for the patient,^39^ a detail which is lost when investigating individual CVDs in isolation. This highlights the need to recognise that not only is CVM common,^5^ but that management of established CVD risk factors is likely to be highly important not only in preventing individual CVDs, but also in preventing and delaying progression of CVM.

### Strengths and limitations

The strengths of this study included the large sample size and number of events, enabling more precise estimation of risk factor associations with CVM outcomes. In the Million Women Study, there was virtually complete follow-up and only a small proportion of missing values for the risk factor variables (<6% for each variable). Despite the possibility of residual confounding, the availability of data for a range of important confounders meant that we could adjust for them in these analyses.

One limitation is that the risk factors were reported at study baseline and may have changed over time. Effect estimates for associations with self-reported BMI and height are unlikely to be strongly biased by reporting errors or changes over time.^40^ However, our study may underestimate the associations of continued current smoking with CVM risk, since many women who smoked at recruitment stopped smoking, and smoking cessation is more common among women who smoked less.^41^ Physical activity and alcohol consumption are moderately well-reported in the Million Women Study, but such behaviours may also change over time, which could result in lower power to detect associations.^15 42 43^

Furthermore, self-reported treatment for diabetes, hypertension and high blood cholesterol were used as proxies for the conditions. High agreement between general practice prescription data and self-reported treatment for diabetes and hypertension was found in the Million Women Study.^44^ However, self-reported treatment for high cholesterol is likely to underestimate the prevalence of high cholesterol because at the time of recruitment (1996–2001), statins were just starting to be prescribed in the UK and the incidence of statin prescription was low, though increasing.^45^

Our findings may not generalise to men due to sex differences in CVD incidence and risk factor associations; nevertheless, our study contributes to the historically limited evidence on cardiovascular risk in women compared with men.^46^

## Conclusion

Associations with known cardiovascular risk factors were especially strong with complex CVM and specific CVM pairs, which may be overlooked when treating patients with individual CVDs in isolation. Our findings suggest that early and intensive risk factor management in line with clinical guidelines for CVD prevention^47^—particularly lifestyle and risk factor modification for obesity, smoking, hypertension and diabetes—might also help prevent multiple downstream cardiovascular events. Moreover, proactive clinical management of cardiovascular risk factors may confer greater benefit for those at risk of developing higher numbers of co-occurring CVDs. Further research is needed to understand whether risk factors for CVM pairs vary by the temporal order of disease occurrence, or for recurrent events of the same CVD subtype, which may inform individualised prevention strategies against CVM.

## Supplementary material

10.1136/openhrt-2025-003950 online supplemental file 1

## Data Availability

Data are available upon reasonable request. Data may be obtained from a third party and are not publicly available.
