# Supplementary material for: Risk factors for multimorbidity of cardiovascular diseases in the prospective Million Women Study
Source: Open Heart. 2026 May 21;13(1):e003950. doi: 10.1136/openhrt-2025-003950 (PMC13202100; doi:10.1136/openhrt-2025-003950)
Supplement: online supplemental file 1 [file openhrt-13-1-s001.docx]

**Supplemental material to**

**Risk factors for multimorbidity of cardiovascular diseases in the prospective Million Women Study**

Contents

[**Appendix A. Selection of cardiovascular disease subtypes** 2](#_Toc216225687)

[**Appendix B. Study flow diagram** 5](#_Toc216225688)

[**Appendix C. Number and proportion of missing values for key characteristics self-reported at recruitment** 6](#_Toc216225689)

[**Appendix D. Risk factor associations in minimally adjusted models** 8](#_Toc216225690)

[**Appendix E. Sensitivity analysis including non-drinkers** 10](#_Toc216225691)

# **Appendix A. Selection of cardiovascular disease subtypes**

Individual cardiovascular disease subtypes (CVDs) to comprise cardiovascular multimorbidity (CVM) were selected using the International Classification of Diseases, 10^th^ Revision (ICD-10) classification system and linked follow-up data in a four-step process to comprehensively capture serious CVDs in the Million Women Study cohort.

In the first step, each three-character code in Chapter IX (Diseases of the Circulatory System) of the ICD-10 was assessed to exclude (1) acute conditions which are not indicative of underlying chronic diseases (e.g. I46: cardiac arrest), (2) common antecedent conditions which are not typically in and of themselves serious disease states (e.g. I10: primary hypertension), and (3) non-specific codes (e.g. I27: other pulmonary heart disease). Non-specific codes that may represent important classes of conditions that could be grouped with a specific condition (e.g. I09: other rheumatic heart diseases) were not excluded.

In the second step, CVDs which are not included in Chapter IX of ICD-10 were identified and selected. Transient cerebral ischaemic attacks and related syndromes (G45), vascular dementia (F01), and vascular disorders of intestine (K55) were included from other chapters of the ICD-10.

In the third step, the three-character ICD-10 codes selected in the first two steps were further aggregated (e.g. I20-I25: ischaemic heart disease) or separated (e.g. I35.0/2 aortic stenosis) to form aetiologically and/or anatomically distinct diseases.

In the fourth and final step, individual CVDs which were relatively common in the Million Women Study cohort were included for investigation as components of CVM in this study. An event was defined as the first hospital admission after recruitment with a relevant ICD-10 code in any position (i.e. primary or secondary diagnosis fields) or death with the diagnosis as the underlying cause, whichever came first. Only events that were recorded after the date of recruitment up to 31 December 2016 were counted. Note that these were not strictly incident events, since participants with prior CVD at recruitment had not been excluded yet. Note also that end of follow-up when the CVDs were selected was 31 December 2016, whereas the analyses presented in this study included additional follow-up time up to 31 December 2019. Nineteen CVDs with 5,000 or more events by the end of 2016 were finally included (Table A1). The selected CVDs could be broadly grouped into three disease classes: heart diseases, cerebrovascular diseases, and other vascular diseases.

We acknowledge that some of the CVD subtypes we have defined may not be clearly distinct aetiologically and/or anatomically. For example, the ICD-10 codes that define ‘other cerebrovascular disease’ encompasses a range of conditions such as ‘other nontraumatic intracranial haemorrhage (I62)’, ‘occlusion and stenosis of precerebral/cerebral arteries, not resulting in cerebral infarction (I65, I66)’ or ‘cerebral disorders in diseases classified elsewhere (I68)’ (e.g. I68.0, cerebral amyloid angiopathy). We acknowledge that these cannot be cleanly separated from stroke and vascular dementia and will largely co-occur with those conditions (which we observe in the data). However, we aimed to include a comprehensive range of CVDs as represented by the ICD-10 coding system and commonly recorded in HES and death records. We therefore chose to include these codes under the umbrella term, ‘other cerebrovascular disease’.

Table A1 List of selected individual cardiovascular diseases, corresponding diagnostic codes, and number of events between study recruitment and 31 December 2016, after standard exclusions only

|  | **ICD-10 code*** | | **Events (N)**** |  |
| --- | --- | --- | --- | --- |
| **Heart diseases** |  |  |  |  |
| Rheumatic heart disease | I05-I09 | | 22,731 |  |
| Hypertensive heart and renal disease | I12-13 | | 14,329 |  |
| Ischaemic heart disease | I20-25 | | 154,991 |  |
| Mitral valve disorders | I34 |  | 16,292 |  |
| Aortic stenosis | 135.0, I35.2 | | 14,801 |  |
| Other aortic valve disorders | I35.1, I35.8, I35.9 | | 6,760 |  |
| Cardiomyopathy | I42 |  | 5,162 |  |
| Other arrhythmias | I44-45, I47, I49 | | 54,398 |  |
| Atrial fibrillation | I48 |  | 91,980 |  |
| Heart failure | I11.0, I13.0, I13.2, I50 | | 50,089 |  |
| **Cerebrovascular diseases** |  |  |  |  |
| Stroke | I60, 61, I63, I64 | | 40,530 |  |
| Transient cerebral ischaemic attacks and related syndromes | G45 |  | 14,918 |  |
| Other cerebrovascular disease | I62, I65-69 | | 40,995 |  |
| Vascular dementia | F01 |  | 7,810 |  |
| **Arterial, venous, and other vascular diseases** |  |  |  |  |
| Venous thromboembolism | I26, I80-82 | | 35,039 |  |
| Aortic aneurysm | I71 |  | 7,487 |  |
| Peripheral vascular disease | I73.9 |  | 17,008 |  |
| Arterial embolism and thrombosis | I74 |  | 5,069 |  |
| Vascular disorders of the intestine | K55 |  | 7,994 |  |
| *Diagnostic codes used to identify diseases from hospital admission and death records  **An event was defined as the first hospital admission after recruitment with a relevant ICD-10 code in any position or death with the diagnosis as the underlying cause, whichever came first. These were not considered incident events, since women with cardiovascular disease prior to recruitment were not excluded from the cohort yet. | | | |  |

# **Appendix B. Study flow diagram**

**
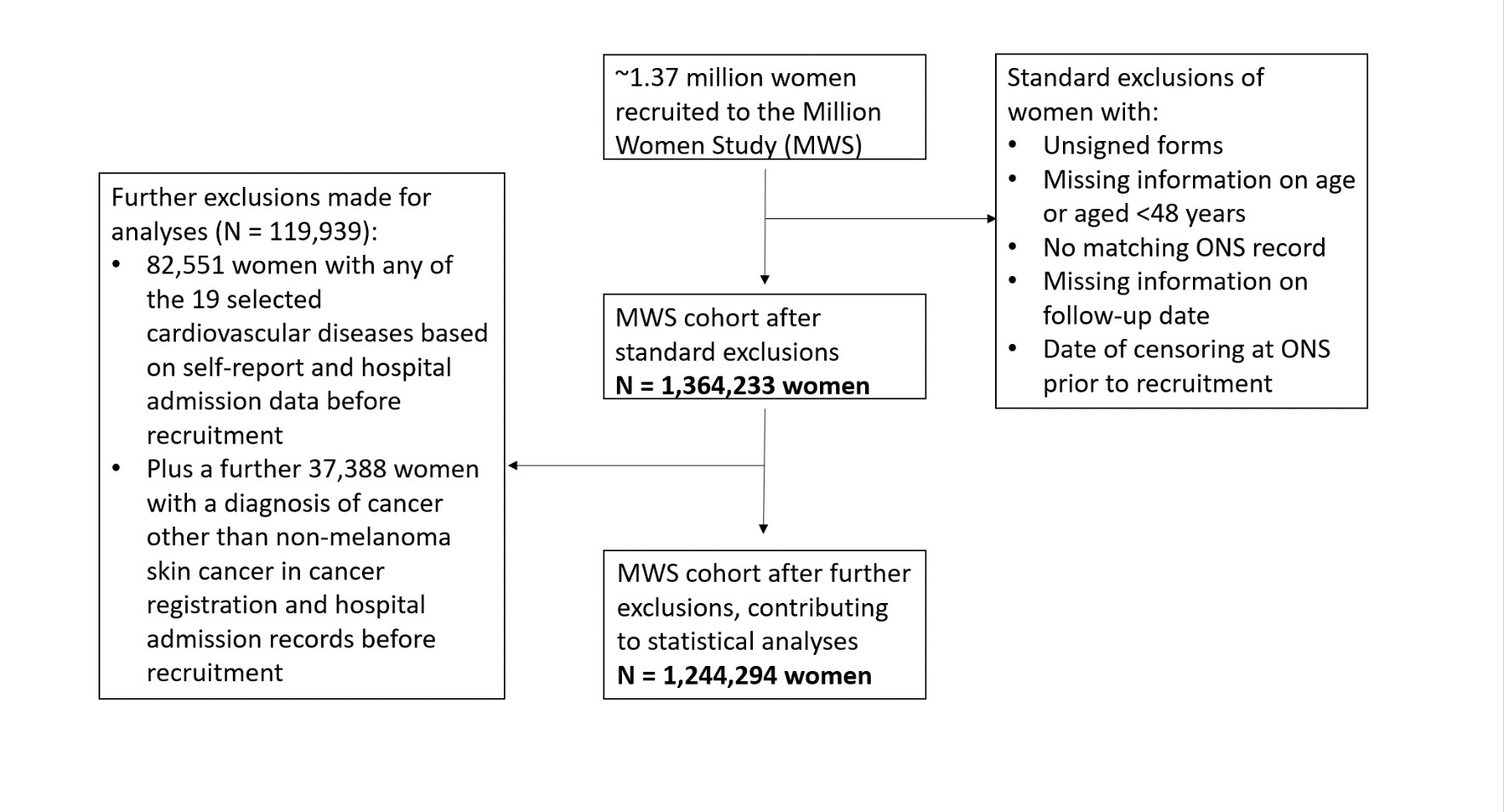
**

# **Appendix C. Number and proportion of missing values for key characteristics self-reported at recruitment**

| All women, n | 1,244,294 |
| --- | --- |
| Established cardiovascular risk factors | % (n) |
| **Smoking** |  |
| Never | 48.7 (605,592) |
| Past | 26.3 (327,285) |
| Current <15 per day | 9.8 (122,302) |
| Current 15+ per day | 9.4 (116,954) |
| Missing | 5.8 (72,161) |
| **Alcohol consumption (units/week)** |  |
| Rarely/Never | 34.7 (431,862) |
| 1-6 | 40.7 (507,023) |
| 7-14 | 18.7 (232,793) |
| 15+ | 5.1 (63,182) |
| Missing | 0.8 (9,434) |
| **Strenuous physical activity** |  |
| Rarely/Never | 46.1 (573,589) |
| Up to once | 29.8 (370,268) |
| 2-3 times | 14.4 (178,992) |
| 4+ times | 6.1 (76,178) |
| Missing | 3.6 (45,267) |
| **Body mass index (kg/m^2^)** |  |
| <20 | 3.9 (48,029) |
| 20-25 | 41.2 (512,743) |
| 25-30 | 33.6 (417,533) |
| 30-35 | 11.6 (144,294) |
| 35+ | 4.6 (57,242) |
| Missing | 5.2 (64,453) |
| **Height (cm)** |  |
| <155 | 17.4 (216,743) |
| 155-159 | 14.9 (184,950) |
| 160-164 | 29.5 (367,659) |
| 165-169 | 22.1 (275,279) |
| 170+ | 14.5 (180,285) |
| Missing | 1.6 (19,378) |
| Morbidities | % (n) |
| **Being treated for hypertension** |  |
| Yes | 14.5 (180,503) |
| No | 85.4 (1,063,116) |
| Missing | 0.1 (675) |
| **Being treated for diabetes mellitus** |  |
| Yes | 2.1 (25,745) |
| No | 97.9 (1,217,769) |
| Missing | 0.1 (780) |
| **Being treated for high blood cholesterol** |  |
| Yes | 2.6 (32,447) |
| No | 85.8 (1,067,745) |
| Missing (including 11.5% who were not asked) | 11.6 (144,102) |
| Socio-economic factors | % (n) |
| **Area deprivation*** |  |
| Least deprived fifth | 20.3 (253,191) |
| 2 | 20.2 (251,309) |
| 3 | 20.0 (248,253) |
| 4 | 19.7 (245,556) |
| Most deprived fifth | 19.0 (236,910) |
| Missing | 0.7 (9,075) |
| **Educational attainment** |  |
| 1 | 13.2 (163,640) |
| 2 | 25.9 (322,365) |
| 3 | 16.4 (203,744) |
| 4 | 41.9 (521,857) |
| 5 | 2.6 (32,688) |
| Missing | 13.2 (163,640) |
| Reproductive factors | % (n) |
| **Parity** |  |
| Nulliparous | 10.8 (134,936) |
| 1-2 | 55.6 (692,056) |
| 3+ | 33.2 (412,741) |
| Missing | 0.4 (4,561) |
| **Ever use of menopausal hormone therapy** |  |
| Never | 49.1 (610,643) |
| Ever | 49.8 (619,389) |
| Missing | 1.1 (14,262) |
| *Fifths of the Townsend deprivation score^[[1]](#footnote-1)^ |  |

# **Appendix D. Risk factor associations in minimally adjusted models**

| Minimally adjusted hazard ratio (95% confidence interval) | | | | | | | | | | | | | | | |  |
| --- | --- | --- | --- | --- | --- | --- | --- | --- | --- | --- | --- | --- | --- | --- | --- | --- |
|  | **Cardiovascular multimorbidity** | | | **Complex cardiovascular multimorbidity** | **Ischaemic heart disease & Atrial fibrillation** | | **Ischaemic heart disease & Heart failure** | | **Atrial fibrillation & Heart failure** | | **Atrial fibrillation & Stroke** | | **Ischaemic heart disease & Stroke** | | **Heart failure & Stroke** |  |
| **BMI (kg/m^2^)** | |  |  | | |  | |  | |  | |  | |  | | |
| <20 | 1.21 (1.18-1.24) | | | 1.18 (1.11-1.25) | 1.06 (1.00-1.14) | | 1.17 (1.09-1.26) | | 1.17 (1.09-1.27) | | 0.99 (0.89-1.10) | | 1.25 (1.13-1.40) | | 1.19 (1.02-1.39) |  |
| 20-24 | 1.00 (referent) | | | 1.00 (referent) | 1.00 (referent) | | 1.00 (referent) | | 1.00 (referent) | | 1.00 (referent) | | 1.00 (referent) | | 1.00 (referent) |  |
| 25-29 | 1.23 (1.22-1.25) | | | 1.28 (1.25-1.32) | 1.31 (1.27-1.34) | | 1.46 (1.41-1.50) | | 1.41 (1.37-1.46) | | 1.18 (1.13-1.23) | | 1.29 (1.23-1.35) | | 1.37 (1.28-1.46) |  |
| 30-34 | 1.71 (1.68-1.74) | | | 1.96 (1.90-2.02) | 1.95 (1.89-2.01) | | 2.42 (2.33-2.51) | | 2.44 (2.35-2.53) | | 1.67 (1.58-1.76) | | 1.82 (1.71-1.93) | | 2.29 (2.12-2.47) |  |
| 35+ | 2.65 (2.59-2.70) | | | 3.35 (3.23-3.48) | 3.22 (3.09-3.35) | | 4.37 (4.19-4.55) | | 5.15 (4.94-5.36) | | 2.55 (2.38-2.73) | | 2.57 (2.38-2.77) | | 4.12 (3.75-4.51) |  |
| **Height (cm)** | |  |  | | |  | |  | |  | |  | |  | | |
| <155 | 1.00 (referent) | | | 1.00 (referent) | 1.00 (referent) | | 1.00 (referent) | | 1.00 (referent) | | 1.00 (referent) | | 1.00 (referent) | | 1.00 (referent) |  |
| 155-159 | 0.96 (0.95-0.98) | | | 0.96 (0.92-0.99) | 1.01 (0.97-1.05) | | 0.91 (0.87-0.95) | | 1.00 (0.96-1.05) | | 0.98 (0.92-1.05) | | 0.92 (0.86-0.98) | | 0.92 (0.84-1.00) |  |
| 160-164 | 0.94 (0.92-0.95) | | | 0.91 (0.89-0.94) | 1.01 (0.98-1.04) | | 0.79 (0.77-0.82) | | 1.01 (0.97-1.05) | | 1.07 (1.01-1.13) | | 0.85 (0.80-0.90) | | 0.85 (0.79-0.91) |  |
| 165-169 | 0.97 (0.96-0.99) | | | 0.96 (0.93-0.99) | 1.11 (1.07-1.15) | | 0.78 (0.75-0.81) | | 1.10 (1.06-1.15) | | 1.15 (1.09-1.22) | | 0.79 (0.75-0.84) | | 0.83 (0.77-0.90) |  |
| 170+ | 1.12 (1.10-1.14) | | | 1.15 (1.11-1.19) | 1.31 (1.26-1.36) | | 0.87 (0.83-0.90) | | 1.46 (1.40-1.52) | | 1.36 (1.28-1.45) | | 0.85 (0.79-0.91) | | 0.95 (0.87-1.04) |  |
| **Smoking** |  | | |  |  | |  | |  | |  | |  | |  |  |
| Never | 1.00 (referent) | | | 1.00 (referent) | 1.00 (referent) | | 1.00 (referent) | | 1.00 (referent) | | 1.00 (referent) | | 1.00 (referent) | | 1.00 (referent) |  |
| Past | 1.24 (1.23-1.26) | | | 1.33 (1.30-1.36) | 1.34 (1.30-1.37) | | 1.42 (1.37-1.46) | | 1.32 (1.29-1.37) | | 1.17 (1.13-1.23) | | 1.28 (1.22-1.35) | | 1.29 (1.21-1.37) |  |
| Current <15 per day | 1.84 (1.81-1.87) | | | 2.16 (2.09-2.23) | 1.75 (1.69-1.81) | | 2.48 (2.39-2.57) | | 1.95 (1.87-2.03) | | 1.59 (1.50-1.69) | | 2.24 (2.11-2.38) | | 2.20 (2.03-2.39) |  |
| Current 15+ per day | 2.58 (2.54-2.62) | | | 3.14 (3.05-3.24) | 2.41 (2.33-2.49) | | 3.72 (3.59-3.86) | | 2.83 (2.72-2.94) | | 2.11 (1.99-2.24) | | 3.36 (3.17-3.56) | | 3.42 (3.16-3.70) |  |
| **Alcohol (units/week)** | |  |  | | |  | |  | |  | |  | |  | | |
| 1 to 2 | 1.00 (referent) | | | 1.00 (referent) | 1.00 (referent) | | 1.00 (referent) | | 1.00 (referent) | | 1.00 (referent) | | 1.00 (referent) | | 1.00 (referent) |  |
| 3 to 6 | 0.95 (0.93-0.96) | | | 0.92 (0.90-0.95) | 0.93 (0.90-0.96) | | 0.90 (0.86-0.93) | | 0.91 (0.88-0.94) | | 0.97 (0.92-1.02) | | 0.90 (0.84-0.96) | | 0.94 (0.86-1.02) |  |
| 7 to 14 | 0.95 (0.94-0.97) | | | 0.92 (0.90-0.95) | 0.93 (0.90-0.96) | | 0.85 (0.82-0.88) | | 0.93 (0.89-0.96) | | 1.03 (0.98-1.08) | | 0.90 (0.84-0.96) | | 0.88 (0.80-0.96) |  |
| 15+ | 1.06 (1.04-1.09) | | | 1.04 (1.00-1.09) | 1.05 (1.00-1.10) | | 0.96 (0.90-1.01) | | 1.17 (1.11-1.23) | | 1.28 (1.19-1.37) | | 1.09 (0.98-1.20) | | 1.06 (0.92-1.21) |  |
| *Continued on the next page* | | | | | | | | | | | | | | | |  |

|  | | | | | | |  | | | | | |  | | | | | | |  | | | | | |
| --- | --- | --- | --- | --- | --- | --- | --- | --- | --- | --- | --- | --- | --- | --- | --- | --- | --- | --- | --- | --- | --- | --- | --- | --- | --- |
| Appendix E (continued) | | | | | | | | |  | | | | | | | | |  | | | | | | | |
| Multivariable adjusted hazard ratio (95% confidence interval) | | | | | | | | | | | | | | | | | | | | | | | | | |
|  | | **Cardiovascular multimorbidity** | | | | **Complex cardiovascular multimorbidity** | | | **Ischaemic heart disease & Atrial fibrillation** | | | **Ischaemic heart disease & Heart failure** | | **Atrial fibrillation & Heart failure** | | | | **Atrial fibrillation & Stroke** | | | **Ischaemic heart disease & Stroke** | | | | **Heart failure & Stroke** |
| **Strenuous physical activity** | | | |  |  | | | | | | | | | | |  | | | | | |  | | | |
| None | | 1.00 (referent) | | | | 1.00 (referent) | | | 1.00 (referent) | | | 1.00 (referent) | | 1.00 (referent) | | | | 1.00 (referent) | | | 1.00 (referent) | | | | 1.00 (referent) |
| Up to once per week | | 0.73 (0.73-0.74) | | | | 0.69 (0.67-0.70) | | | 0.72 (0.70-0.74) | | | 0.60 (0.58-0.62) | | 0.66 (0.64-0.68) | | | | 0.79 (0.76-0.83) | | | 0.69 (0.66-0.73) | | | | 0.62 (0.59-0.67) |
| 2-3 times per week | | 0.69 (0.68-0.70) | | | | 0.65 (0.63-0.68) | | | 0.69 (0.67-0.71) | | | 0.54 (0.52-0.56) | | 0.62 (0.60-0.65) | | | | 0.76 (0.72-0.81) | | | 0.63 (0.60-0.68) | | | | 0.58 (0.54-0.64) |
| 4+ times per week | | 0.80 (0.78-0.82) | | | | 0.78 (0.75-0.82) | | | 0.80 (0.76-0.84) | | | 0.72 (0.69-0.76) | | 0.74 (0.70-0.78) | | | | 0.86 (0.79-0.93) | | | 0.79 (0.72-0.86) | | | | 0.79 (0.71-0.88) |
| **Being treated for high blood pressure** | | |  | | | | |  | | |  | | | |  | | | |  | | | |  | | |
| No | | 1.00 (referent) | | | | 1.00 (referent) | | | 1.00 (referent) | | | 1.00 (referent) | | 1.00 (referent) | | | | 1.00 (referent) | | | 1.00 (referent) | | | | 1.00 (referent) |
| Yes | | 1.84 (1.82-1.87) | | | | 2.19 (2.15-2.24) | | | 2.16 (2.11-2.21) | | | 2.15 (2.10-2.21) | | 2.26 (2.20-2.32) | | | | 2.40 (2.31-2.49) | | | 2.28 (2.18-2.38) | | | | 2.52 (2.38-2.66) |
| **Being treated for diabetes** |  | | | | | |  | | |  | | |  | | | |  | | |  | | | |  | |
| No | | 1.00 (referent) | | | | 1.00 (referent) | | | 1.00 (referent) | | | 1.00 (referent) | | 1.00 (referent) | | | | 1.00 (referent) | | | 1.00 (referent) | | | | 1.00 (referent) |
| Yes | | 3.17 (3.10-3.24) | | | | 4.30 (4.14-4.46) | | | 3.13 (2.99-3.27) | | | 5.81 (5.59-6.04) | | 3.62 (3.45-3.80) | | | | 2.61 (2.42-2.82) | | | 4.41 (4.11-4.73) | | | | 5.46 (5.01-5.94) |
| **Being treated for high blood cholesterol** | | |  | | | | |  | | |  | | | |  | | | |  | | | |  | | |
| No | | 1.00 (referent) | | | | 1.00 (referent) | | | 1.00 (referent) | | | 1.00 (referent) | | 1.00 (referent) | | | | 1.00 (referent) | | | 1.00 (referent) | | | | 1.00 (referent) |
| Yes | | 1.56 (1.52-1.60) | | | | 1.75 (1.67-1.82) | | | 1.67 (1.59-1.75) | | | 1.80 (1.71-1.89) | | 1.39 (1.31-1.47) | | | | 1.22 (1.12-1.33) | | | 1.90 (1.75-2.06) | | | | 1.53 (1.37-1.72) |
| Models were stratified by age at recruitment and area of residence | | | | | | | | |  | | | | | | | | |  | | | | | | | |

# **Appendix E. Sensitivity analysis including non-drinkers**

Relative risks of incident cardiovascular multimorbidity and complex cardiovascular multimorbidity in relation to alcohol consumption, including non-drinkers as a sensitivity analysis

| **Outcome:** | **Cardiovascular multimorbidity** | **Complex cardiovascular multimorbidity** |
| --- | --- | --- |
| Alcohol (units/week) | HR (95% CI) | HR (95% CI) |
| Rarely/never | 1.14 (1.12-1.16) | 1.15 (1.11-1.18) |
| 1 to 2 | 1.00 (referent) | 1.00 (referent) |
| 3 to 6 | 0.95 (0.93-0.96) | 0.94 (0.90-0.97) |
| 7 to 14 | 0.94 (0.92-0.96) | 0.91 (0.87-0.94) |
| 15+ | 1.01 (0.98-1.04) | 0.99 (0.93-1.05) |

Multivariable adjusted models were stratified by age at recruitment and area of residence and additionally adjusted for area deprivation,

educational attainment, physical activity, smoking, height, body mass index, parity, and ever use of menopausal hormone therapy.

1. Townsend P, Phillimore P, Beattie A. Health and Deprivation: Inequality and the North (1st ed.): Routledge; 1988. [↑](#footnote-ref-1)
